# Rational Incorporation of Selenium into Temozolomide Elicits Superior Antitumor Activity Associated with Both Apoptotic and Autophagic Cell Death

**DOI:** 10.1371/journal.pone.0035104

**Published:** 2012-04-05

**Authors:** Yan Cheng, Ugir Hossain Sk, Yi Zhang, Xingcong Ren, Li Zhang, Kathryn J. Huber-Keener, Yuan-Wan Sun, Jason Liao, Shantu Amin, Arun K. Sharma, Jin-Ming Yang

**Affiliations:** Department of Pharmacology and The Penn State Hershey Cancer Institute, The Pennsylvania State University College of Medicine and Milton S. Hershey Medical Center, Hershey, Pennsylvania, United States of America; Wayne State University School of Medicine, United States of America

## Abstract

**Background:**

The DNA alkylating agent temozolomide (TMZ) is widely used in the treatment of human malignancies such as glioma and melanoma. On the basis of previous structure-activity studies, we recently synthesized a new TMZ selenium analog by rationally introducing an *N*-ethylselenocyanate extension to the amide functionality in TMZ structure.

**Principal Findings:**

This TMZ-Se analog showed a superior cytotoxicity to TMZ in human glioma and melanoma cells and a more potent tumor-inhibiting activity than TMZ in mouse glioma and melanoma xenograft model. TMZ-Se was also effective against a TMZ-resistant glioma cell line. To explore the mechanism underlying the superior antitumor activity of TMZ-Se, we compared the effects of TMZ and TMZ-Se on apoptosis and autophagy. Apoptosis was significantly increased in tumor cells treated with TMZ-Se in comparison to those treated with TMZ. TMZ-Se also triggered greater autophagic response, as compared with TMZ, and suppressing autophagy partly rescued cell death induced by TMZ-Se, indicating that TMZ-Se-triggered autophagy contributed to cell death. Although mRNA level of the key autophagy gene, *Beclin 1*, was increased, Beclin 1 protein was down-regulated in the cells treated with TMZ-Se. The decrease in Beclin 1 following TMZ-Se treatment were rescued by the calpain inhibitors and the calpain-mediated degradation of Beclin1 had no effect on autophagy but promoted apoptosis in cells treated with TMZ-Se.

**Conclusions:**

Our study indicates that incorporation of Se into TMZ can render greater potency to this chemotherapeutic drug.

## Introduction

TMZ is an oral DNA alkylating agent currently used as an upfront treatment for glioblastoma multiforme, the most common and aggressive type of primary brain tumor in human. This chemotherapeutic agent is also utilized for treating patients with metastatic melanoma; in particular, due to its ability to penetrate through the blood-brain barrier, TMZ is often prescribed to patients with brain metastases of melanoma [1]. This drug exerts its antitumor activity by interfering with DNA replication. TMZ can methylate DNA leading to formation of O^6^-methylguanine, the most potent cell-killing lesion, which mispairs with thymine during the next cycle of DNA replication. Subsequent futile cell cycles of DNA mismatch result in DNA double strand breaks (DSBs) and trigger cell death [2]. Although TMZ can improve overall survival of patients with cancer, the therapeutic outcomes of this therapy remain unsatisfactory. For instance, nearly 3/4 of the TMZ-treated glioma patients still die within 2 years, and as many as 50% of brain tumors are TMZ-resistant. Thus, developing more effective anticancer drugs would help improve the outcome of treatment of the malignancies such as glioblastoma multiforme and melanoma.

**Figure 1:**
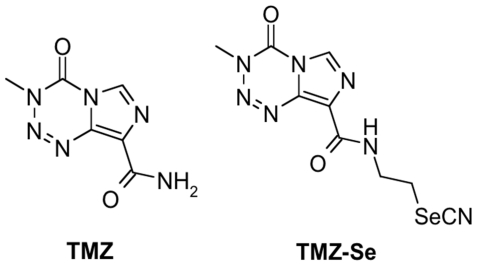
Chemical structures of TMZ-Se and TMZ.

The rationale for introducing selenium into the molecule is that the tredox-active trace element, selenium, is an essential micro-nutrient and a normal component of diets with chemopreventive and anticancer properties [3]. Various forms of selenium compounds have been shown to possess apoptosis-inducing and anti-proliferative effects, and are thus considered as potential chemopreventive and chemotherapeutic agents for cancer [4], [5], [6]. In this study, we tested whether incorporating selenium into TMZ would improve the anticancer properties of this drug. TMZ exhibits its anticancer effects by rapid chemical conversion in the systemic circulation at physiological pH to the active compound monomethyl triazeno imidazole carboxamide (MTIC), which further degrades to amino imidazole carboxamide (AIC). The optimization of the TMZ structure was therefore performed in such a way as not to hinder this mechanism, which apparently is responsible for 100% bioavailability of TMZ. We synthesized a TMZ selenium derivative (TMZ-Se) by rationally introducing an *N*-ethylselenocyanate extension to the amide functionality known to be uninvolved in TMZ metabolism, which is responsible for its activity, thus creating an agent that should contain the properties of both TMZ and selenium. The chemical structure of TMZ-Se is shown in Fig. 1. Our results show that TMZ-Se not only showed superior cytotoxicity to TMZ in human glioma and melanoma cells, but also was effective against TMZ-resistant tumor cells. In mouse melanoma and glioma xenograft models, TMZ-Se also demonstrated stronger anti-tumor activity. The greater cytotoxicity of TMZ-Se may be attributed to its stronger inducing effects on DSBs, apoptosis and autophagy.

## Results

### Introduction of Selenium into TMZ Produces Superior Cytotoxicity to TMZ in Human Glioma and Melanoma Cells

**Figure 2:**
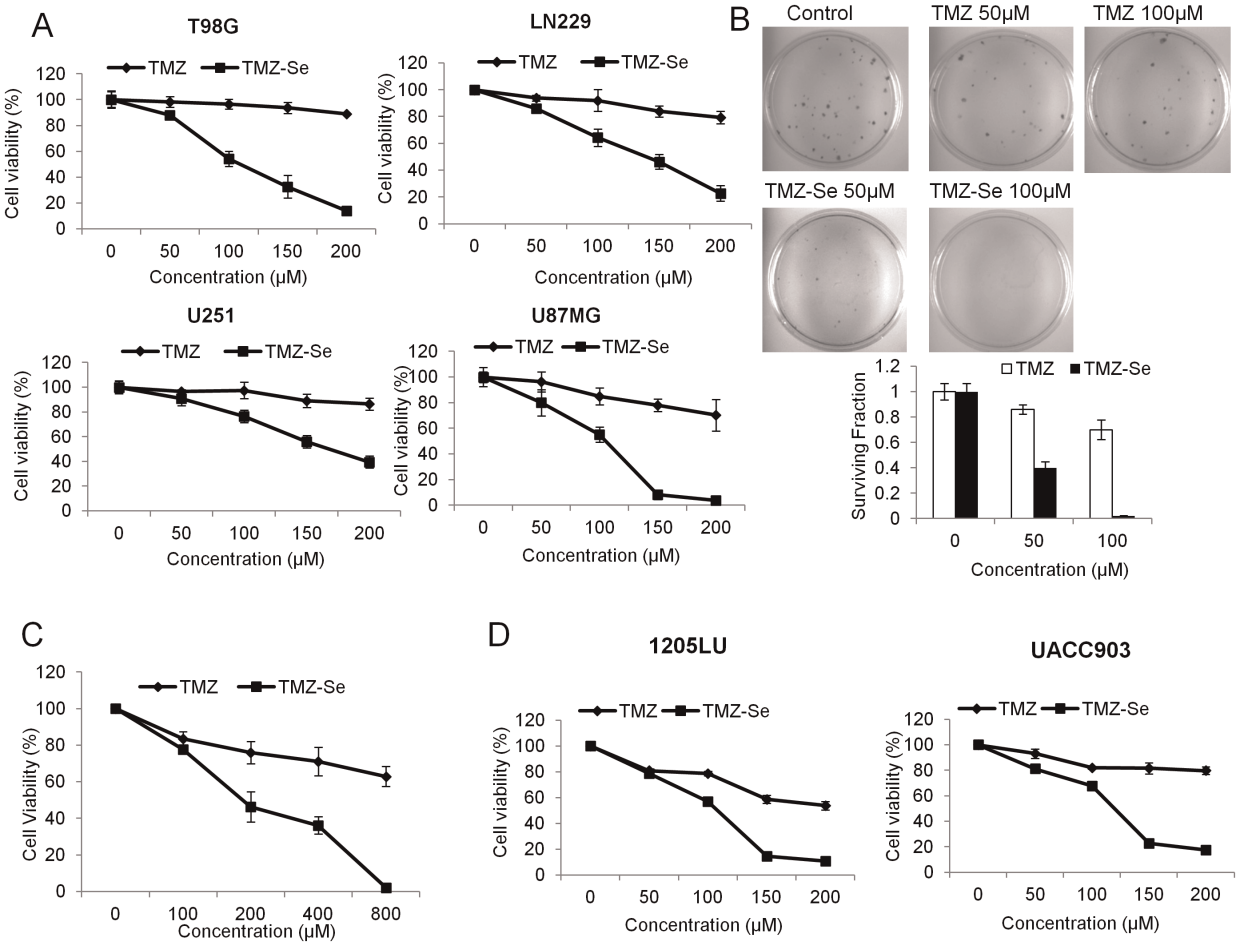
Comparison of cytotoxicity of TMZ-Se and TMZ in glioma and melanoma cell lines. (**A**) Human glioma cell lines T98G, LN229, U251 and U87MG were treated with a series concentration of TMZ or TMZ-Se for 48 h, and cell viability was measured by MTT assay. (**B**) T98G cells were treated with TMZ or TMZ-Se for 3 days, and then were incubated for 12 days at 37°C in a humidified atmosphere containing 5% CO_2_/95% air. At the end of incubation, the cells were stained with 1% methylene blue in 50% methanol for 30 min, washed with water, and colonies counted. (**C**) TMZ-resistant glioma cell line CCF-STTG was treated with TMZ or TMZ-Se for 72 h, and cell viability was measured by MTT assay. (**D**) Human melanoma cell lines 1205LU and UACC903 were treated with a series concentration of TMZ or TMZ-Se for 48 h, and cell viability was measured by MTT assay.

To determine whether our newly developed TMZ/selenium derivative, TMZ-Se, had better activity than TMZ in killing tumor cells, we compared the cytotoxicity of TMZ and TMZ-Se in four human glioma cell lines T98G, LN229, U251 and U87MG. Fig. 2A shows that TMZ-Se had a greater inhibitory effect on proliferation of the tumor cells than TMZ, as determined by cell viability assay. Colonogenic assay also demonstrated the superior cytotoxicity of TMZ-Se to TMZ in glioma cells (Fig. 2B). Additionally, TMZ-Se showed efficacy in inhibiting cell proliferation in a TMZ-resistant human glioma cell line, CCF-STTG (Fig. 2C). Similar greater cytotoxic effect of TMZ-Se than TMZ was observed in human melanoma cell lines 1205LU and UACC903 (Fig. 2D). These results indicate that the modification of TMZ structure by incorporation of selenium produces superior cytotoxicity to TMZ in malignant tumor cells.

### TMZ-Se Causes a Greater DNA Damage Response and a More Severe Impairment of mTOR Signaling

**Figure 3:**
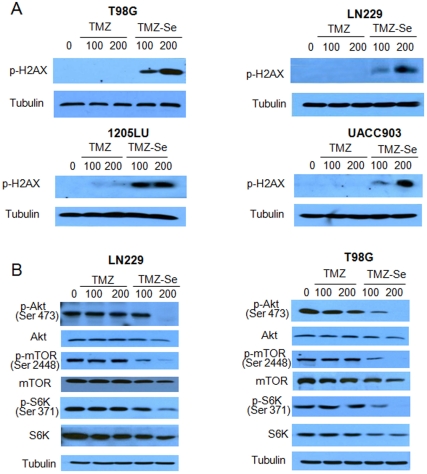
Effects of TMZ-Se and TMZ on phosphorylation of H2AX, and on activity of Akt-mTOR-S6K pathway. (**A**) T98G, LN229, 1205LU and UACC903 cells were treated with 100 µM or 200 µM of TMZ or TMZ-Se for 48h, and the level of phospho-H2AX was measured by Western blot. Tubulin was used as a loading control. (**B**) LN229 and T98G cells were treated with TMZ or TMZ-Se for 48 h, and the levels of p-Akt, Akt, p-mTOR, mTOR, p-S6K, and S6K were measured by Western blot analysis. Tubulin was used as a loading control.

As the cytotoxicity of TMZ is known to be associated with O^6^MeG lesions, which cause DSBs, we compared the level of phospho-histone H2AX, an indicator of DSBs, in glioma and melanoma cells treated with TMZ or TMZ-Se. Fig. 3A shows that the level of phospho-H2AX was remarkably higher in tumor cells treated with TMZ-Se than in cells treated with TMZ, indicating that TMZ-Se treatment causes more DSBs than TMZ. As DNA damage can also cause impairment of mTOR signaling [7] and TMZ was reported to alter mTOR signaling [8] , we compared the effects of TMZ-Se and TMZ on activity of the Akt-mTOR-S6 kinase pathway. As shown in Fig. 3B, the greater DNA-damaging effect of TMZ-Se was also evidenced by the decreases of activity of Akt, mTOR, and S6 kinase, as demonstrated by decreased levels of phospho-Akt, phospho-mTOR, and phospho-S6 kinase.

### TMZ-Se is More Apoptogenic than TMZ in Tumor Cells

**Figure 4:**
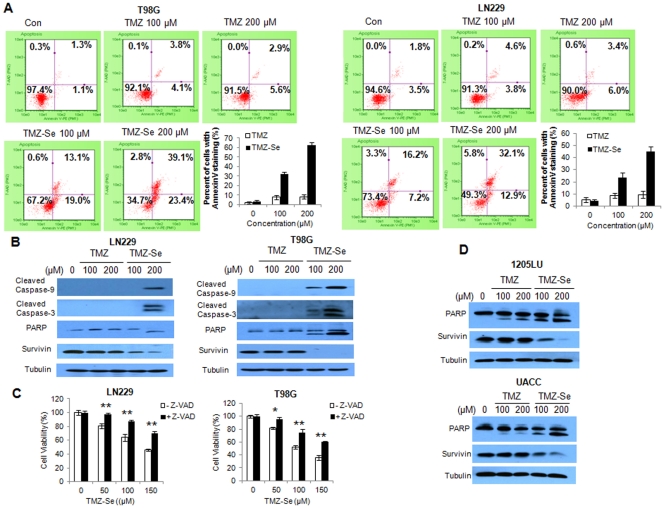
TMZ-Se is more apoptogenic than TMZ in tumor cells. (**A**) LN229 and T98G cells were treated with 100 µM or 200 µM of TMZ or TMZ-Se for 48 h, and apoptosis was examined by flow cytometric analysis of Annexin V and 7-AAD staining. (**B**) LN229 and T98G cells were treated with TMZ or TMZ-Se for 48h, and the levels of caspse-9, caspase-3, PARP and survivin were measured by Western blot analysis. Tubulin was used as a loading control. (**C**) LN229 and T98G cells were treated with TMZ-Se for 48h in the absence or presence of Z-VAD, and cell viability was measured by MTT assay. (**D**) 1205LU and UACC cells were treated with TMZ or TMZ-Se for 48h, and the levels of PARP and survivin were measured by Western blot. Tubulin was used as a loading control. *p < 0.05; **p < 0.01.

Formation of DNA DSBs can trigger apoptosis; therefore, we wanted to know whether TMZ-Se would induce greater apoptosis than TMZ. Flow cytometric analysis of Annexin V staining showed that TMZ-Se caused 3 ∼ 7-fold more apoptosis than TMZ in T98G and LN229 cells, and this apoptogenic effect was dose - dependent (Fig. 4A). Stronger apoptogenic effect of TMZ-Se was validated by the increased amounts of cleaved caspase-3, caspase-9 and PARP, and the decreased amounts of the anti-apoptotic protein, survivin, in the TMZ-Se-treated glioma cells, as determined by western blot (Fig. 4B). To confirm the role of apoptosis in the cell killing caused by TMZ-Se, we measured the cytotoxicity of TMZ-Se in the presence of *pan*-caspase inhibitor, Z-VAD. Fig. 4C shows that the presence of Z-VAD significantly reduced the cytotoxic effect of TMZ-Se in tumor cells, further supporting the role of apoptosis in the cytotoxicity of TMZ-Se. The stronger effects of TMZ-Se on apoptosis were also observed in melanoma cells (Fig. 4D). These results indicate that TMZ-Se possesses greater apoptosis-promoting activity than TMZ.

### TMZ-Se Induces Greater Autophagic Response than TMZ in Tumor Cells

**Figure 5:**
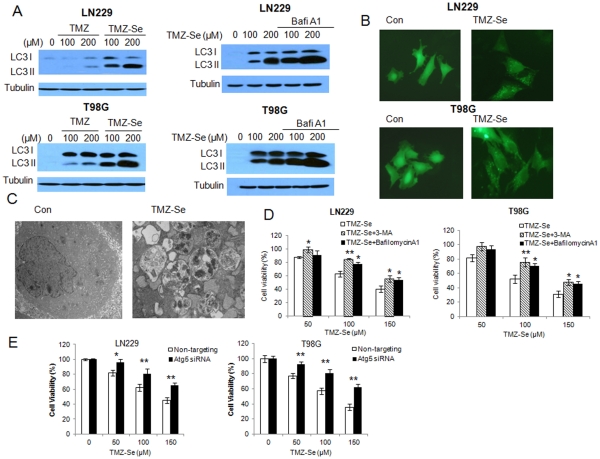
TMZ-Se triggers a greater autophagic response than TMZ, and inhibition of autophagy decreases the efficacy of TMZ-Se against glioma cells. (**A**) *Left panels*: LN229 and T98G cells were treated with TMZ or TMZ-Se for 48 h, and the level of LC3 was measured by Western blot analysis. Tubulin was used as a loading control. *Right panels*: LN229 and T98G cells were treated with TMZ-Se for 48 h in the presence or absence of bafilomycinA1, and the level of LC3 was measured by Western blot analysis. Tubulin was used as a loading control. (**B**) LN229 and T98G cells were transfected with a GFP-LC3 plasmid, followed by treatment with TMZ-Se for 48h. At the end of treatment, the cells were observed under fluorescence microscope. (**C**) T98G cells treated with TMZ-Se or vehicle were harvested by trypsinization, fixed and embedded in spur resin. Ninety nm thin sections were cut and examined at 80 Kv with a JEOL 1200EX transmission electron microscope. (**D**) LN229 and T98G cells were treated with TMZ-Se for 48h in the absence or presence of 3-MA or bafilomycinA1, and cell viability was measured by MTT assay. (**E**) LN229 and T98G cells were transfected with an Atg5-targeted siRNA, and then treated with TMZ-Se for 48h. Cell viability was measured by MTT assay. *p < 0.05; **p < 0.01.

TMZ has been reported to induce autophagy in tumor cells [9], and activation of this cellular process can affect sensitivity of tumor cells to treatments such as chemotherapy and radiotherapy [10]. Thus, we examined and compared autophagenic effects of TMZ-Se and TMZ in tumor cells. As shown in Fig. 5, in comparison to TMZ, TMZ-Se induced greater autophagic response, as evidenced by elevations of the autophagy marker LC3 II (Fig. 5A), increases in the GFP-LC3 dots (Fig. 5B), and accumulation of autophagosomes (Fig. 5C). In the cells treated with TMZ-Se, LC3 II level was further accumulated in the presence of bafilomycinA1, an inhibitor of autophagosome-lysosome fusion and LC3II degradation, indicating that autophagic flux was also enhanced by TMZ-Se (Fig. 5A, right panels). As autophagy can either promote cell survival or death, we further evaluate the role of TMZ-Se-activated autophagy in modulating sensitivity of tumor cells to this new compound. We treated the tumor cells with TMZ-Se in the absence or presence of the autophagy inhibitors, 3-MA, bafilomycinA1 or Atg5 siRNA, and then measured cell viability. Fig. 5D and 5E shows that the cytotoxicity of TMZ-Se against T98G and LN229 cells was significantly decreased when autophagy was suppressed, suggesting that activation of autophagy contributes to the cytocidal action of TMZ-Se.

### TMZ-Se Stimulates Calpain-mediated Degradation of Beclin 1

**Figure 6:**
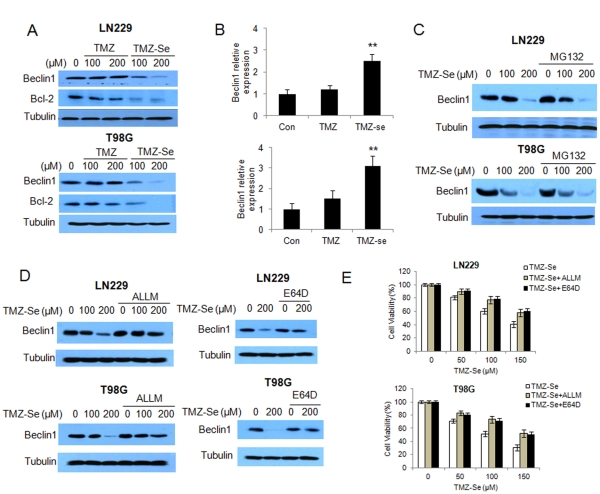
TMZ-Se induces calpain-mediated degradation of Beclin 1. LN229 and T98G cells were treated with 100 or 200 µM of TMZ or TMZ-Se for 48h, (**A**) the levels of Beclin 1 and Bcl-2 were measured by Western blot. Tubulin was used as a loading control; (**B**) the expression of *beclin1* mRNA was measured by qRT-PCR. (**C**) LN229 and T98G cells were treated with 100 or 200 µM of TMZ-Se for 48h in the presence or absence of 10µM of MG132, and the level of Beclin 1 were measured by Western blot. Tubulin was used as a loading control. (**D**) LN229 and T98G cells were treated with TMZ-Se for 48h in the presence or absence of 20µM ALLM (*left panel*) or 10µg/ml E64D (*right panel*), and the level of Beclin1 was measured by Western blot. Tubulin was used as a loading control. (**E**) LN229 and T98G cells were treated with TMZ-Se for 48h in the presence or absence of 20µM ALLM or 10µg/ml E64D, and cell viability was measured by MTT assay. **p < 0.01.

Despite that treatment with TMZ-Se activated autophagy in tumor cells (Fig. 5), we unexpectedly found that the expression of Beclin 1, a key regulator of autophagy, was down-regulated in tumor cells treated with this compound, as examined by Western blot (Fig. 6A). With the down-regulation of Beclin 1, there was also a concomitant decrease in Bcl-2, an anti-apoptotic protein that interacts with Beclin1 (Fig. 6A). Contrastingly, expression of beclin 1 mRNA was increased following TMZ-Se treatment, as analyzed by qRT-PCR (Fig. 6B). These results raised the possibility that down-regulation of Beclin 1 caused by TMZ-Se may result from enhanced degradation of this protein. To test this hypothesis, we first determined whether or not the down-regulation of Beclin 1 protein by TMZ-Se could be reversed by MG132, a proteasomal inhibitor. We found that in spite of the presence of MG132, the TMZ-Se-induced down-regulation of Beclin 1 remained (Fig. 6C), suggesting that the turnover of Beclin 1 is not mediated via the ubiquin-proteasome pathway. Notably, the calpain inhibitors, ALLM and E64D, showed a rescuing effect on Beclin 1 protein in the cells treated with TMZ-Se (Fig. 6D). These results suggest that TMZ-Se may have a stimulatory effect on calpain activity, thus promoting degradation of Beclin 1. We then measured the effects of calpain on cytotoxic activity of TMZ-Se against tumor cells, and found that the cytotoxicity of TMZ-Se was significantly decreased in the presence of ALLM or E64D (Fig. 6E), suggesting that calpain was involved in the TMZ-Se-induced cell death.

**Figure 7:**
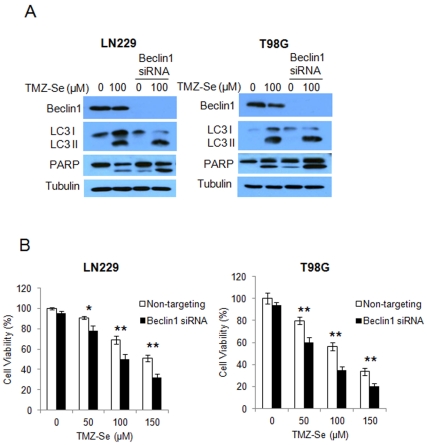
TMZ-Se induces calpain-mediated degradation of Beclin 1. (**A**) LN229 and T98G cells were transfected with Beclin1siRNA followed by TMZ-Se treatment, and the levels of LC3, Beclin 1 and PARP were examined by Western blot. Tubulin was used as a loading control. (**B**) LN229 and T98G cells were transfected with a Beclin 1-targeted siRNA, followed by treatment with TMZ-Se for 48h. Cell viability was measured by MTT assay. *p < 0.05; **p < 0.01.

The concurrent activation of autophagy and down-regulation of Beclin 1 made us query whether TMZ-Se-induced autophagy was Beclin1-independent. We found that in the cells with silencing of Beclin 1 expression, TMZ-Se still caused an increase in LC3 II (Fig. 7A), indicating that dysfunction of Beclin 1 fails to block the activation of autophagy by TMZ-Se, and the TMZ-Se-activated autophagy is indeed Beclin 1-independent. Down-regulation of Beclin 1 appears to contribute to augmentation of apoptosis induced by TMZ-Se, as silencing of Beclin 1 expression further increased the amount of cleavage of PARP (Fig.7A). To further verify the effect of Beclin1 on cell death induced by TMZ-Se, we compared TMZ-Se toxicity in cells with or without silencing of Beclin 1 expression. As shown in Fig. 7B, knockdown of Beclin 1enhanced the sensitivity of tumor cells to TMZ-Se. These results suggest that the degradation of Beclin 1 by calpain plays a role in promoting apoptosis and enhancing cell death in tumor cells treated with TMZ-Se.

### TMZ-Se Shows a Greater Antitumor Activity than TMZ in Glioma and Melanoma Xenograft Models

**Figure 8:**
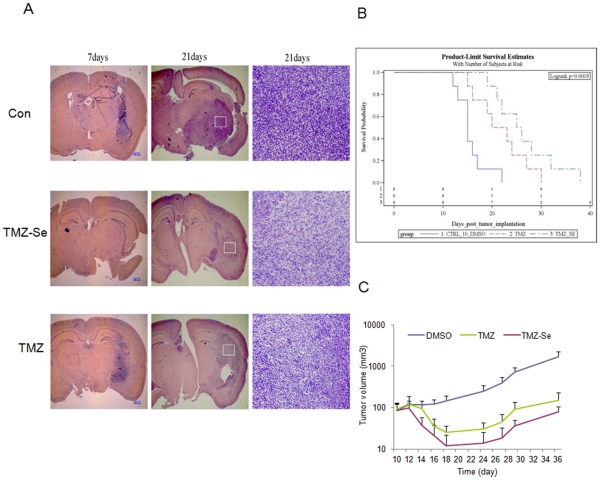
Effects of TMZ-Se and TMZ on tumor growth in mouse glioma and melanoma xenograft models. (**A and B**) The human glioma cells LN229 (1×10^5^ cells in 15 µl of DMEM medium) were injected into the brains of 6-week-old male BALB/c nude mice at 4 mm depth under anesthesia with chloralic hydras (4%, 2ml/kg, ip). Three days after tumor cell implantation, mice were randomly divided into three groups (15 mice/group). Treatments were begun on day 4. TMZ-SE (15 mg/kg), TMZ (15 mg/kg) or vehicle (10% DMSO in saline) was given p.o. daily for 2 weeks. (**A**) At day 7 and day 21 after tumor cell implantation, the mice were euthanized, and the brains were fixed in 10% buffered formalin, embedded in paraffin, and then stained with hematoxillin-eosin (H&E). The images shown are the representative of 5 mice from each group; (**B**) The kaplan-Meier survival curves, n = 10; (**C**) Nude mice (swiss^nu/nu^) were inoculated s.c. with UACC903 human melanoma cells (1×10^6^ cells/100 µl/mouse). When the tumors reached 50∼100 mm^3^ in volume, TMZ or TMZ-Se (15 mg/kg) was administered i.p. on days 1, 3, 5, 7 and 9. Tumor sizes and body weight of the animals were measured every other day. The differences between treatments were analyzed using a two-sample *t*-test. The survival curves of the tumor - bearing mice subjected to different treatments were estimated using Kaplan-Meier method and compared by log-rank statistic analysis.

To test and compare the *in vivo* therapeutic efficacy of TMZ-Se and TMZ, we determined the anti-tumor effects of these two compounds in mice bearing either glioma or melanoma xenografts. In the intracranial LN-229 glioma model, tumor-bearing mice treated with TMZ-Se (15 mg/kg) had longer survival times (Fig. 8B) and smaller tumor masses than those treated with same dose of TMZ, as evidenced by histologic examinations of the brain tissues on day 7 and day 21 following tumor inoculations (Fig. 8A). In a melanoma xenograft model, TMZ-Se also showed a significantly stronger tumor-inhibiting activity as compared to TMZ without observable systemic toxicity (Fig. 8C). On average, the tumor volume is ∼30 mm^3^ lower in TMZ-Se group than in TMZ group (*p*-value = 0.0003). These results indicate that TMZ-Se also had a greater antitumor activity than TMZ *in vivo*, and demonstrate the potential of TMZ-Se as a more effective anticancer agent than TMZ.

## Discussion

Several types of malignant tumors, such as glioblastoma multiforme and melanoma, are notoriously resistant to conventional chemotherapy. Even for TMZ, the frontline drug for treatment of glioblastoma multiforme and metastatic melanoma, its therapeutic outcomes are often disappointing. In the current study, we show that TMZ-Se, a new TMZ analog developed and synthesized by our group, is superior to TMZ in inhibiting viability of glioma and melanoma cells (Fig. 2), and in suppressing growth of tumor xenograts in animal models (Fig. 8). Noteworthily, TMZ-Se also showed efficacy against TMZ-resistant tumor cells (Fig. 2). The cytotoxic effect of methylating agents is believed to result from formation of O^6^-MeG, which causes DNA DSBs that act as a trigger of apoptotic cell death [2]. Also, a number of studies have shown that selenium has a potent anti-cancer activity that correlates with its apoptosis - inducing effects [11], [12]. These observations have recently led to development of several organoselenium small molecule compounds, both in our laboratories and elsewhere, which showed promising anti-tuomr properties [13], [14], [15], [16], [17], [18], [19]. The introduction of selenium into TMZ structure was particularly challenging because the changes in structure should not compromise its ability to cross the blood brain barrier (BBB). Therefore, we designed TMZ-Se by extending the amide functionality in TMZ so as not to disturb the ring involved in degradation to active metabolites and responsible for 100% bioavailability and apparently the BBB crossing ability of TMZ [20]. The -SeCN group was used as carrier of selenium to generate a second active moiety into TMZ structure since it is known to be efficiently metabolized to a selenol (-SeH) intermediate that is responsible for the redox cycling and anticancer properties of selenium. Our results suggest that TMZ-Se acts by retaining the properties of both TMZ and Se. The efficient reduction in brain tumor xenografts clearly indicates that TMZ-Se crosses the BBB when administered orally. This suggests that TMZ-Se metabolizes similar to TMZ, leading to active intermediates by cleaving the tetrazene ring,_which apparently is responsible for its crossing through the BBB. In addition, the enhanced cytotoxicity of TMZ-Se as compared to TMZ indicates the expected usual functioning of -SeCN group, which would cleave to generate a free selenol (-SeH) thus adding the redox cycling properties of Se and enhancing selective cytotoxicity to tumor cells.

We demonstrated that rational introduction of selenium into TMZ greatly increased the inducing effect on DNA DSB (Fig. 3) and on apoptosis (Fig. 4). TMZ-induced apoptosis is a late response that needs at least two cell cycles after treatment [21]. For instance, apoptosis in gliomas is only visible 4–6 days following TMZ treatment [9], and TMZ - treated melanoma cells start to undergo apoptosis 72 h later [22]. It appears that TMZ-Se not only possesses stronger apoptosis - inducing activity but also triggers cell death more rapidly than TMZ, as at 48 h post-treatment, more marked apoptosis were observed in tumor cells treated with TMZ-Se than those treated with TMZ (Fig. 4).

Autophagy is a catabolic process by which damaged organelles and proteins are sequestered into autophagosome and subsequently degraded through fusion with lysosomes. It was found in both glioma cells and tissues that TMZ treatment caused activation of autophagy [9] [23], but the precise roles of autophagy in determining the efficacy of this drug remains uncertain. We found that our new compound, TMZ-Se, induced stronger autophagic response than TMZ in glioma cells (Fig. 5). There has long been debate over whether autophagy is cell-killing or cell-protective in cancers. Autophagy was originally recognized as a cell-protective mechanism under various stressful conditions, but later was also found to play a death-promoting role, causing autophagic cell death [24]. Here, we demonstrated that inhibition of autophagy decreased the efficacy of TMZ-Se against glioma (Fig. 5D and E), indicating that autophagy plays a pro-death role and contributes to the antitumor effects of TMZ-Se. Interestingly, we found that Beclin 1, a critical regulator of autophagy, was decreased in cells treated with TMZ-Se (Fig. 6A), and inhibition of calpain, a Ca^2+^-dependent protease involved in control of cell proliferation and apoptosis, blocked the degradation of Beclin1 induced by TMZ-Se (Fig. 6D). Calpain-triggered degradation of Beclin 1 has been reported by Yoo BH *et al*. who demonstrated that calpain activity is required for Ras-dependent down-regulation of Beclin-1 [25]. Additionally, we found that the decrease in Beclin 1 protein was not accompanied by a reduction in LC3 but resulted in an increase in PARP (Fig. 7A), suggesting that TMZ-Se triggers a non-canonical, Beclin 1-independent autophagy, and the calpain-mediated degradation of Beclin 1 promotes the apoptotic response to this agent. It was previously reported that Beclin 1 participates in inhibition of apoptosis, and that silencing of Beclin 1 expression augments the mitochondrial permeabilization and apoptosis induced by Fas stimulation or doxorubicin treatment in tumor cells [26]. The previous studies also demonstrated that the interaction of Beclin 1 with either Bcl-2 or Bcl-XL is essential for the anti-apoptotic effects of Beclin 1 [27]. We have reported that inhibition of Beclin 1 increased the caspase activities in glioma cells treated with TRAIL via reducing survivin level [28]. Here, we observed a decrease in Bcl-2 expression in glioma cells subjected to TMZ-Se treatment, indicating that the decrease of Beclin 1 leads to the dissociation of Beclin1 with Bcl-2, thereby promoting the degradation of Bcl-2 and causing apoptosis. All of these results suggest that there might be new mechanisms of action involved in the antitumor effects of TMZ-se.

TMZ-Se also showed a stronger antitumor activity in both the glioma and melanoma mouse models (Fig. 8), suggesting that TMZ selenium derivatives may hold promise for further developing as novel chemotherapeutic agents. We are planning further pre-clinical studies including pharmacokinetic comparisons to determine whether or not TMZ-Se warrants clinical trials in the hope of developing it to a useful and effective anticancer agent.

Taken together, by introducing selenium into TMZ, we have developed a novel TMZ analog that demonstrates superior anti-tumor activity against glioma and melanoma both *in vitro* and *in vivo*. This also suggests that by modifying the amide function, we were able to retain the BBB-penetrating capability of Se-TMZ while enhancing its tumor-inhibitory property. We believe that appropriately incorporating selenium into certain conventional anticancer drugs to improve their effectiveness might represent a new strategy of drug development that is worth further investigation.

## Materials and Methods

### Synthesis of TMZ-Se

The 4-methyl-5-oxo-2,3,4,6,8-pentazabicyclo[4.3.0]nona-2,7,9-triene-9-(*N*-selenocyanatoethyl) carboxamide (TMZ-Se) was synthesized starting from TMZ according to our recently developed method (manuscript under preparation). Briefly, the amide functionality in TMZ was first converted to an acid chloride by treating TMZ with sodium nitrite followed by hydrolysis to give the corresponding acid, and subsequently treating with thionyl chloride. Treatment of acid chloride with aminoethyl bromide gave bromo intermediate that was finally converted to the desired TMZ-Se by a reaction with potassium selenocyanide. The compound was characterized by NMR and MS and its purity (>99%) was determined by HPLC.

### Cell Lines and Culture

The human glioblastoma cell lines, T98G, LN-229, U251 and U87MG, and human melanoma cell lines 1205LU and UACC were purchased from American Type Culture Collection (ATCC). T98G cells were cultured in Ham’s F-10: DMEM (10∶1) medium, and LN-229, U251, U87MG, 1205LU and UACC cells were cultured in DMEM medium. These media were supplemented with 10% fetal bovine serum, 100 units/mL penicillin, and 100 µg/mL streptomycin. Cells were maintained at 37°C in a humidified atmosphere containing 5% CO_2_/95% air. All cultures were monitored routinely and found to be free of contamination by mycoplasma or fungi. All cell lines were discarded after three months and new lines propagated from frozen stocks.

### Reagents and Antibodies

TMZ was purchased from Tocris Bioscience (Ellisville, Missouri). Anti-phospho-H2AX antibody was purchased from upstate-cell signaling solutions (Lake placid, New York). 3-(4, 5-dimethylthiazol-2-yl)-2,5-diphenyltetrazolium bromide (MTT), 3-methyladenosine (3-MA), MG132, ALLM, bafilomycin A1 and Z-VAD were purchased from Sigma (Denver, Colorado). Anti-caspase-3, anti-caspase-9, anti-PARP, anti-survivin, anti–LC-3, anti–α-tubulin, anti -Akt, anti-phospho-Akt (Ser 473), anti–phospho-mTOR (Ser 2448), anti–mTOR, anti–phospho-S6 kinase (Ser371), anti–S6 kinase, anti-Beclin1 antibodies were purchased from Cell Signaling Technologies (Boston, Massachusetts). All cell culture media and other reagents were purchased from Invitrogen (Carlsbad, California). Western blot reagents were obtained from Pierce Biotechnology (Rockford, Illinois) .

### Measurement of Autophagy

Autophagy was monitored using the following methods: 1) Western blot analysis of LC3; 2) microscopic observation of GFP-LC3 puncta; 3) electron microscopic examination of double or multi-membrane vacuoles in the cytoplasm.

### Western Blot

Cells were lysed in M-PER mammalian protein extraction reagent (Thermo Scientific, Rochester, New York) supplemented with a protease inhibitor cocktail (Roche, South San Francisco, California) at room temperature for 5 minutes followed by centrifugation at 14,000×g for 10 minutes. Protein Concentrations of the cell lysates were measured using the Bio-Rad DC assay (Bio-Rad, Hercules, California). Proteins (20–40 µg) were resolved on SDS-PAGE and transferred to PVDF membrane (Bio-Rad, Hercules, California). The blots were incubated with indicated antibodies in 3% BSA/TBST at 4°C for overnight followed by incubation with secondary antibodies at room temperature for 1 h. The protein signals were detected by ECL method.

### Apoptosis Assays

Apoptosis was determined by flow cytometric analysis of Annexin V and 7-AAD staining. Briefly, 100 µl Guava Nexin reagent (Millipore, Billerica, Massachusetts) was added to 1×10^5^ cells (in 100 µl) and the cells were incubated with the reagent for 20 min at room temperature in the dark. At the end of incubation, the cells were analyzed by a Guava EasyCyte™Plus Flow Cytometry System (Millipore, Billerica, Massachusetts).

### Cellular Viability Assay

Cell viability was measured by MTT assay. Briefly, cells were plated at 5×10^3^ cells per well in 96-well tissue culture plates and incubated at 37°C in a humidified atmosphere containing 5% CO_2_/95% air. The formazan product, formed after 4h incubation with MTT, was dissolved in DMSO and read at 570 nm on a Victor3 Multi Label plate reader (PerkinElmer, San Jose, California).

### Clonogenic Assay

Tumor cells were plated in 35-mm cell culture dishes (200 cells/dish) and treated with drug for 3 days. Then, the cells were incubated at 37°C in a humidified atmosphere containing 5% CO_2_/95% air for 12days. At the end of the incubation period, cells were stained with 1% methylene blue in 50% methanol for 30 min, washed with water, and colonies counted.

### Real Time-PCR

Total RNAs were isolated from the treated cells using the Trizol isolation reagent (Roche, South San Francisco, California) according to the manufacturer’s instruction. The concentrations of total RNAs were measured using a NanoDrop (Thermo Scientific, Rochester, New York). First-strand cDNA was synthesized using Omniscript reverse transcription kit (Qiagen, Valencia, California) with random primers, according to the manufacturer’s instruction. Real time PCR was performed on a Stratagene Mx3005P using Brilliant II SYBR Green QPCR master mix (Stratagene, La Jolla, California) and following primer sets: *beclin1*, 5′-CAA GAT CCT GGA CCG TGT CA-3′ (forward) and 5′-TGG CAC TTT CTG TGG ACA TCA-3′ (reverse); *β-actin*, 5′-GCC AAC ACA GTG CTG TCT GG-3′ (forward) and 5′-GCT CAG GAG GAG CAA TGA TCT TG-3′ (reverse). After 40 cycles, data were collected and analyzed by MxPro software (Stratagene, La Jolla, California).

### Animal Study

*Orthotopic glioblastoma mouse model:* Six-week old male BALB/c nude mice were used for intracerebral injections of glioma cells. Mice were anesthetized with chloralic hydras (4%, 0.2ml/100g, i.p.), and then were inoculated with LN229 cells (1×0^5^) in 15 µl of DMEM in the brains at 4.3 mm depth. Three days after tumor implantation, mice were randomly divided into three groups (15 mice/group). Treatment was initiated on day 4. TMZ-Se (15 mg/kg), TMZ (15 mg/kg) or vehicle were given p.o. daily for 2 weeks. Animal body weight and physical sign were monitored during experiment. Five mice of each group were killed at day 7 and 21 following tumor inoculation. Brains were fixed in 10% buffered formalin and embedded in paraffin, and then stained with hematoxillin-eosin (H&E). *Melanoma mouse model:* Nude mice (swiss^nu/nu^) were inoculated s.c. with UACC903 melanoma cells (1×10^6^ cells/100 µl/mouse). When the tumors reached 50∼100 mm^3^ in volume, the mice were treated i.p. with TMZ or TMZ-Se (15 mg/kg) on days 1, 3, 5, 7 and 9. Tumor sizes and body weight of the animals were measured every other day. Mice were housed in an animal facility and were maintained in a temperature-controlled and light-controlled environment. The animal experiments were approved by the Institutional Animal Care and Use Committee of Penn State College of Medicine. The differences between treatments were analyzed using a two-sample *t*-test. The survival curves of the tumor - bearing mice subjected to different treatments were estimated using Kaplan-Meier method and compared by log-rank statistic analysis.
